# How Much *g* Is in the Distractor? Re-Thinking Item-Analysis of Multiple-Choice Items

**DOI:** 10.3390/jintelligence8010011

**Published:** 2020-03-09

**Authors:** Boris Forthmann, Natalie Förster, Birgit Schütze, Karin Hebbecker, Janis Flessner, Martin T. Peters, Elmar Souvignier

**Affiliations:** Institute of Psychology in Education, University of Münster, 48149 Münster, Germany; natalie.foerster@uni-muenster.de (N.F.); birgit.schuetze@uni-muenster.de (B.S.); karin.hebbecker@uni-muenster.de (K.H.); flessner@uni-muenster.de (J.F.); martin.peters@uni-muenster.de (M.T.P.); elmar.souvignier@uni-muenster.de (E.S.)

**Keywords:** Raven’s progressive matrices, intelligence, distractors, item analysis

## Abstract

Distractors might display discriminatory power with respect to the construct of interest (e.g., intelligence), which was shown in recent applications of nested logit models to the short-form of Raven’s progressive matrices and other reasoning tests. In this vein, a simulation study was carried out to examine two effect size measures (i.e., a variant of Cohen’s ω and the canonical correlation *R_CC_*) for their potential to detect distractors with ability-related discriminatory power. The simulation design was adopted to item selection scenarios relying on rather small sample sizes (e.g., *N* = 100 or *N* = 200). Both suggested effect size measures (Cohen’s ω only when based on two ability groups) yielded acceptable to conservative type-I-error rates, whereas, the canonical correlation outperformed Cohen’s ω in terms of empirical power. The simulation results further suggest that an effect size threshold of 0.30 is more appropriate as compared to more lenient (0.10) or stricter thresholds (0.50). The suggested item-analysis procedure is illustrated with an analysis of twelve Raven’s progressive matrices items in a sample of *N* = 499 participants. Finally, strategies for item selection for cognitive ability tests with the goal of scaling by means of nested logit models are discussed.

## 1. Introduction

Distractors are a fundamental part of the item content in multiple-choice items (Thissen et al. 1989; Guttman and Schlesinger 1967). That fact is taken into account in both traditional and contemporary distractor analysis (Gierl et al. 2017). An approach that falls in the category of contemporary distractor analysis is Myszkowski and Storme’s (2018) nested logit model application to the latest short form of Raven’s Progressive Matrices. The nested logit model family (Suh and Bolt 2010) concurrently uses accuracy and distractor choice information from each item to improve ability estimation. That is, item responses to multiple-choice items are modeled in terms of solution behavior (i.e., solved vs. not-solved) by means of a logistic item response theory (IRT) model for binary items (e.g., 1PL, 2PL or 3PL) at the first place. Then, given the item has not been solved, distractor choices are modeled by Bock’s nominal response model (NRM) (Bock 1972). Hence, nested logit models, as used by Myszkowski and Storme (2018), include varying discrimination parameters for each distractor. Traditional distractor analysis, as part of a thorough item analysis, does not necessarily focus on this aspect of distractor choices.

The primary focus on solution behavior and a secondary focus on distractor choices is one advantage of nested logit models for the modeling of figural matrix items as compared to other polytomous IRT models. Indeed, there is clear evidence in the literature that using constructive matching [i.e., a strategy focused on constructing the correct solution (Snow 1980; Bethell-Fox et al. 1984)] is positively correlated with cognitive ability. This was found for constructive matching indicators (e.g., self-reported strategy use, estimated latent strategy classes, or the proportion of overall time spent on the item content) derived from the paperfolding test (Snow 1980), figural analogies (Bethell-Fox et al. 1984; Schiano et al. 1989), and figural matrices (Vigneau et al. 2006; Mitchum and Kelley 2010; Hayes et al. 2011; Gonthier and Thomassin 2015; Gonthier and Roulin 2019). In addition, analogous indicators for usage of distractor elimination strategies (e.g., the proportion of overall time spent on the response alternatives or back and forth eye movements between the item content and the response alternatives) were found to be negatively correlated with test performance (Bethell-Fox et al. 1984; Schiano et al. 1989; Vigneau et al. 2006; Hayes et al. 2011; Jarosz and Wiley 2012; Arendasy and Sommer 2013; Gonthier and Thomassin 2015; Gonthier and Roulin 2019). In line with these findings, (Myszkowski and Storme 2018) pointed out that nested logit models which take into account solution behavior, as well as distractor choice, are perhaps best suited to model solution processes starting with constructive matching, and given that a solution, cannot be reached, shifting towards distractor elimination strategies at a later stage. Indeed, Gonthier and Roulin (2019) reported results in line with the idea that both constructive matching and response elimination might be used on the same item.

In addition, the idea that distractor choice provides useful psychometric information existed even before the invention of IRT models for polytomous scoring (Guttman and Schlesinger 1967; Davis and Fifer 1959), and hence, informativeness of distractors has been studied by other approaches than IRT. This is evident in early studies that examined gender in relation to certain error patterns, such as failing to discriminate between the correct option and a distractor designed by rotating the correct solution (Sigel 1963; Vejleskov 1968). In a similar vein, Jacobs and Vandeventer (1970) found that the proportion of choosing distractors that either take into account solely the horizontal or solely the vertical facet was positively correlated with performance on the Coloured Progressive Matrices. Moreover, Vodegel Matzen et al. (1994) found that choosing distractors that share features with the solution or distractors that were a repetition of one of the adjacent entries to the missing element in the matrix discriminated best between children with varying levels of performance [for a complete overview of studies focusing on error analysis in figural matrix items see Kunda et al. (2016)]. Finally, IRT approaches were also found to reveal discriminatory power of distractors with respect to ability (Myszkowski and Storme 2018; Thissen 1976; Storme et al. 2019).

To sum up, evidence on strategy use and informativeness of distractors in figural matrix items seemingly adhere to the idea behind nested logit models. Hence, in combination with the use of rule-based distractor generation (Guttman and Schlesinger 1967; Hornke and Habon 1986; Matzen et al. 2010; Blum et al. 2016; Blum and Holling 2018) to construct items with discriminating distractors, this item family appears to be promising for test development based on nested logit models. In particular, this allows the construction of tests with higher measurement precision at the lower end of the ability range because differentiated information about the ability of those who did not solve the item is taken from distractor choices (Myszkowski and Storme 2018; Storme et al. 2019). To date, however, proper distractor evaluation tools for such item development are not available. In addition, direct use of nested logit models with small sample sizes (e.g., *N* = 200) seems not feasible in light of the many parameters that need to be estimated. In this vein, it is especially unclear which descriptive statistics are informative to allow item pre-selection based on criteria in line with the idea of distractor discrimination at an early stage in the item selection process when candidate items are tested in small samples. Thus, the goal of the current work is to examine Cohen’s ω (Cohen 1992) based on ability groups and distractor choice and the canonical correlation (Thompson 1984; Klecka 1980) between test performance and distractor choice for their potential to detect items with discriminatory distractors. First, we review the potential of traditional distractor analysis tools to reveal the discriminatory power of an item’s distractor set and propose Cohen’s ω and the canonical correlation as useful effect sizes that correspond with similar approaches found in the literature. Second, we evaluate how well these effect sizes perform as a detection method for item pre-selection in a simulation study. Finally, we apply the proposed effect sizes for distractor analysis to the dataset around which this special issue is organized to evaluate the distractors’ potential to be used for nested logit models.

### 1.1. Distractor Analysis as Part of Traditional Analysis of Multiple-Choice Items

Item analysis refers to a set of descriptive statistics that are useful during the process of developing an item pool for a new psychological test (e.g., for the measurement of intelligence). These statistics are most often used in pilot studies in which the item pool (or a subset thereof) is administered to a relatively small sample (say *N* = 50 to *N* = 300) for the purpose of informed item selection. In this process, item analysis is used to provide the first evidence of each item’s psychometric properties, such as difficulty, dimensionality, or discrimination (Henrysson 1971). In this work, we will focus on item discrimination which refers to the relationship between a person’s ability and item performance (Lord 1980; Yen and Fitzpatrick 2006). A highly discriminating item results in a higher solving probability for persons with higher ability level and in lower solving probability for persons with lower ability level (i.e., as compared to a low discriminating item in which solving probabilities are more evenly distributed across the range of ability levels). More specifically, we will focus on ability-related discriminatory power of distractors in multiple-choice items, but not at the level of solution behavior (i.e., accuracy). That is, in case that an item has not been solved correctly by a test-taker it might be the case that choosing a particular distractor vs. choosing one of the other distractors is more likely for persons with higher ability, whereas, persons with lower ability are less likely to choose this distractor. Distractor discrimination parameters are indeed included in certain IRT models, such as the NRM or nested logit models. However, here we focus on more simple item effect sizes that can potentially reveal if items have discriminative distractors in pilot studies which usually have small sample sizes. Pilot studies for scale development can have goals of varying complexity. For example, the smallest sample sizes have been proposed for very early checks of instruction or item wordings (Johanson and Brooks 2010). Pilot studies with a focus on more complex properties of psychological tests, such as latent variable profiles (Von der Embse et al. 2014), for example, may have even sample sizes as large as 1000 participants. For the purpose of this work, we consider reasonably large sample sizes that reflect practice in cognitive ability research (Arendasy et al. 2006). On this basis, items can be selected for a test that, in the next step, could be scaled by means of a nested logit model. This would increase the reliability for low ability test takers (Myszkowski and Storme 2018; Suh and Bolt 2010; Storme et al. 2019).

Perhaps the best-known index of discrimination in item analysis is the item-scale correlation (Henrysson 1971; Cureton 1966) and its various corrections for part-whole overlap (Henrysson 1971) or lack of reliability (Cureton 1966). Conceptually, the same information is captured by factor loadings in factor analytical methods (Henrysson 1962) and discrimination parameters included in IRT models, such as the 2PL to 4PL models (Barton and Lord 1981) or the generalized partial credit model (Muraki 1992), for example. Hence, simple item-scale correlations can be considered as the pilot study counterpart of model parameters included in more complex approaches used for the final scaling of the test. For traditional item analysis, cut-offs exist to decide if items from a pilot study are retained in the item pool for final test calibration. Several suggestions for such cut-offs have been made in the literature, such as item-scale correlations >0.30 (Nunnally and Bernstein 1994) or at least >0.20 (Crocker and Algina 1986). In addition, similar cut-offs exist for standardized factor loadings (Kline 2000) taken from factor analytical approaches which are also used in pilot studies. However, comparable complementary sets of item statistics and model parameters for item selection and final scaling of the test, respectively, along with commonly used item-scale correlation cut-offs for item selection are not available for items with potentially discriminative distractors. 

To illustrate traditional distractor analysis statistics, we created two example datasets, and used the first item from each of these datasets. To facilitate illustration below, we simply refer to the first item from the first dataset to as Example-Item 1 and to the first item from the second dataset to as Example-Item 2. These two items were simulated with three distractors and according to the same population model despite the distractor discrimination parameters. For both items, correct solution behavior was modeled by means of the 2PL with moderate discrimination and difficulty parameters ranging from −1.15 to −0.85 for a set of *difficult* items (see the setup for the simulation study in Section 2.1.2). When participants did not solve the item, their distractor choice was simulated according to NRM intercept parameters as described below, and for Example-item 1 the discrimination parameters were fixed at zero, whereas, for Example-Item 2, high discrimination parameters were simulated. Software code to replicate these data are available in the Open Science Framework repository for this work (https://osf.io/9tp8h/). All statistics introduced below are illustrated for these two items in Table 1 and interpreted in more detail in the respective sections and subsections below.

#### 1.1.1. Distractor Choice Frequency

The distractor choice frequency is often applied as the first criterion for distractor evaluation with the recommendation that useful distractors should be chosen by at least 5% of the participants (Haladyna and Downing 1993). Distractors not fulfilling this criterion in a pilot study would be considered as non-functioning and need revision. Hence, we conclude that distractors in pilot studies should in the first place pass the 5% frequency criterion before subjecting them to an evaluation of their potential to discriminate individuals with respect to the target latent trait. For Example-item 1 and Example-item 2, there were no distractors with choice frequencies below 5%.

#### 1.1.2. The Point-Biserial Correlation

Several indexes have been suggested that connect test performance (i.e., ability estimates) with distractor choice. Perhaps, the most popular index is the point-biserial correlation *PB_D_* (Gierl et al. 2017; Attali and Fraenkel 2000) that contrasts test performance between participants who chose distractor *D* with the participants who did not choose *D* (i.e., the participants who chose either the correct solution or one of the other distractors):

(1) $$PB_{D}=\frac{M_{D}-M}{S}\sqrt{\frac{P_{D}}{1-P_{D}}}.$$

*M_D_* is the average performance of participants who chose *D*, *M* is the average performance of all participants, *S* is the standard deviation of the performance of all participants, and *P_D_* is the proportion of participants who selected *D*. Well-functioning distractors show a negative *PB_D_* (Attali and Fraenkel 2000). However, Attali and Fraenkel (2000) pointed out that the groups of participants contrasted by *PB_D_* do not yield the relevant information for developers in every situation. For example, a positive *PB_D_* can be found for rather difficult items even when the average score of participants choosing *D* is substantially lower than the average score of participants solving the item (i.e., *M* is also affected by participants who chose one of the other distractors). Hence, they suggest an alternative index *PB_DC_* that contrasts the group choosing *D* only with the group who solved the item:

(2) $$PB_{DC}=\frac{M_{D}-M_{DC}}{S_{DC}}\sqrt{\frac{P_{D}}{P_{C}}}.$$

*M_DC_* is the average sum correct score of the participants who either chose *D* or the correct solution *C*, *S_DC_* is the standard deviation of the sum correct score of the group choosing either *D* or *C*, and *P_C_* is the proportion of participants choosing the correct solution. It is clear that this index provides better contrast between distractor choice and item solution in terms of ability. However, both contrasts are not informative for the aim of detecting distractors with discriminatory power with respect to ability because this would require a contrast between participants choosing distractor *D* and participants choosing any other distractor. For Example-item 1 and Example-item 2, there were on average no differences observable for both *PB_D_* and *PB_DC_*. This is indeed expected given that both indices focus on a different aspect of discrimination as compared to the distractor discrimination parameters in nested logit models (see Table 1).

However, a corresponding variant of the point-biserial correlation is possible and could be calculated for each distractor, but this approach has two disadvantages: (a) Given that only participants who did not solve the item would be contrasted, such an index would be more prone to lacking empirical substance (i.e., very small group sizes for some of the distractor contrasts), and (b) looking at as many effect sizes as there are distractors in an item is expected to suffer from cumulative type-I-error (i.e., selecting an item for its discriminatory distractors when the true model behind the item has zero to negligible distractor discrimination). Consequently, we propose a different approach in Section 1.2 that circumvents these issues and relies on effect sizes at the item level.

#### 1.1.3. Trace Line Plots and χ^2^ Statistics

**Figure 1:**
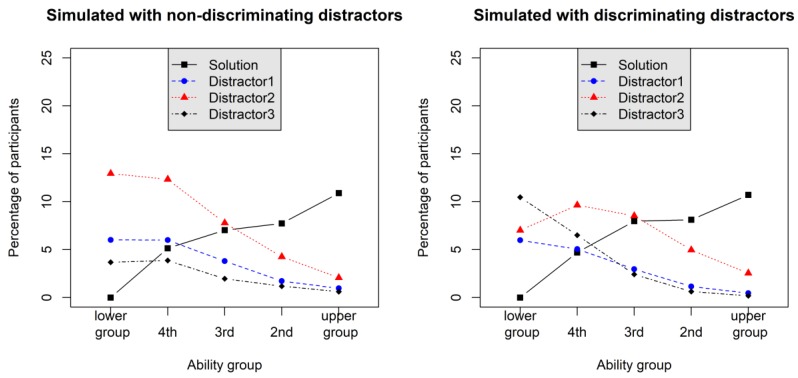
Trace line plots (Gierl et al. 2017; Wainer 1989) of two simulated items (*N* = 10,000 each) when distractors were simulated as non-discriminating in a 2PL (left plot) and when distractors were simulated with moderate NRM (nominal response model) discrimination in a 2PNL.

An alternative index connecting the ability with distractor choice extends a graphical tool labeled option characteristic curve by Levine and Drasgow (1983)—also known as option trace lines as it was labeled by Wainer (1989) or simply trace line plot (Gierl et al. 2017). For instance, choice frequency is plotted as a function of ability groups based on raw scores and the item options (i.e., the correct solution and each of the distractors). Figure 1 displays two trace line plots for Example-item 1 and Example-item 2. Clearly, in both plots, frequency of choosing the correct option was a positive function of ability (we used five ability groups here) with nearly the same trace line. For the distractors on the left side, it can be seen that they possessed varying attractiveness for the participants. Furthermore, distractor choice was a monotonically decreasing function of ability for all distractors. Haladyna and Downing (1993) suggested a $\chi_{D}^{2}$ statistic for each distractor to test whether distractor choice frequencies follow a uniform distribution across ability groups.

However, this statistic again focuses on each distractor in separation from the others and does not reveal anything about an interaction effect between distractor choice and ability group, which would be the crucial characteristic of the discriminatory power of distractors. This is also highlighted by Cohen’s ω results based on the Haladyna-Downing approach (explicitly labelled ω*_D_* to distinguish it from the effect size measure ω*_G_* that will be introduced below) for Example-Item 1 and Example-Item 2 (see Table 1). On average ω*_D_* was comparable in this case. One might argue that the variation of ω*_D_* across distractors could be sensitive for the discriminatory power of distractors, but using a dispersion index (e.g., *SD* of ω*_D_* across distractors) would yield a measure with a rather non-intuitive metric (as compared to commonly used effect size metrics). Thus, in this work, we aim at a direct effect size quantification of the discriminatory power of distractors. In addition, there were no intersections of the trace lines for the distractors between the ability groups in the left plot of Figure 1 (Example-Item 1). The $\chi_{D}^{2}$ statistic, however, would nonetheless be significant for all distractors in this plot (see also the large ω*_D_* values in Table 1). In this vein, it has been conjectured by Garcia-Perez (2014) that non-monotonic empirical trace lines are required (such as those ones depicted for Example-Item 2 in the right plot of Figure 1) to allow effective modeling by polytomous IRT models. Hence, effect sizes are needed, which are sensitive for the detection of distractor trace lines that display distractor-ability interaction effects. In this work, we will use an effect size based on the χ^2^ statistic using ability groups (as shown in Figure 1). In this approach, however, all distractors are considered (i.e., participants solving an item will be discarded from analysis); for a comparable implementation see Levine and Drasgow (1983). However, they used a less intuitive metric as the one that we will introduce in Section 1.2.

#### 1.1.4. Rising Selection Ratios

Love (1997) suggested the criterion of rising selection ratios as a basic property of multiple-choice tests subjected to polytomous scoring. This criterion implies that the odds for choosing the correct option vs. distractor *D* is a monotone increasing function of ability. It is noteworthy, that this criterion does not require relative frequency of choosing *D* to be a monotone decreasing function of ability, because the probability of choosing the correct option relative to choosing *D* (i.e., the criterion of rising selection ratios) can be fulfilled with choice frequencies of *D* being a non-monotone function of ability [see Revuelta (2005) for applying this criterion to the 3PL and various polytomous IRT models]. Hence, primarily, the criterion of rising selection ratios is in accordance with modeling accuracy in the first place as a function of ability as it is put forth in nested logit models. At the same time, this criterion allows for interactions between ability and distractor choice behavior, due to the non-monotonicity of distractor choice in ability. Love (1997) suggested to use Goodman and Kruskal’s γ coefficient (Goodman and Kruskal 1979) between test performance calculated by the sum total scores on all the other items (i.e., not the item under consideration for testing rising selection ratios) and the probability estimated from a 2 × *J* contingency table with *J* as the number of possible test performance scores. The two rows include the frequencies for choosing the correct option and the frequencies for choosing *D* and the probability for choosing probability is then calculated by dividing the entries in the correct-option row by the respective column sums (Love 1997). However, this approach to evaluate data for rising selection ratios does not tap into potential interaction effects between ability and distractor choice. This is illustrated by the findings in Table 1. Goodman-Kruskal γ was found to be 1 for every distractor in Example-item 1 and Example-item 2, and hence, was not sensitive to the difference between these items in terms of distractor discrimination.

### 1.2. Effect Sizes for the Detection of Discriminatory Distractors

#### 1.2.1. Cohen’s ω Based on Ability Groups and Distractor Choice

We suggest Cohen’s ω effect size based on the χ^2^ derived from a contingency table in which the rows represent the distractors and the columns represent ability groups. Hence, this effect size is analogous to the above-mentioned approach used by Levine and Drasgow (1983), but it has a normed range that is easier to interpret. They also scale the χ^2^ statistic for better interpretability. In particular, the χ^2^ should be independent of the number of participants who did not solve the item under consideration because the raw χ^2^ statistic would clearly depend on item difficulty otherwise (Levine and Drasgow 1983). Cohen’s ω (Cohen 1992) can be calculated by

(3) $$\omega_{G}=\sqrt{\frac{\chi_{G}^{2}}{\sum_{i=1}^{k}N_{D_{k}}}}.$$

*G* is the number of ability groups (e.g., as built by quantiles), $\chi_{G}^{2}$ is the χ^2^ statistic based on the *G* ability groups and all *K* distractors, and $\overset{k}{\underset{i=1}{\sum }}N_{D_{k}}$ is the number of all participants who did not solve the item under consideration. In this study, we will examine Cohen’s well-known interpretation guideline for ω*_G_* (Cohen 1992). Specifically, we will use 0.10 (small effect size), 0.30 (medium effect size), and 0.50 (large effect size) as cut-offs for the detection of items with discriminatory distractor sets. Example-Item 1 had ω_2_ = 0.02 and ω_5_ = 0.04, whereas, Example-Item 2 had ω_2_ = 0.24 and ω_5_ = 0.34. This illustrates that the discriminatory power of distractors could potentially be detected with this variant of Cohen’s ω.

#### 1.2.2. Canonical Correlation Based on Ability and Distractor Choice

Coefficient η has been suggested by Haladyna (2004) to be indicative of discriminatory power of item distractors. Accordingly, distractors with comparable choice means (implying a rather small η coefficient) render an item potentially less suitable for polytomous scoring as compared to an item with varying choice means. However, for reasons of a better conceptual fit, we will shift away from the η coefficient to the canonical correlation coefficient. The canonical correlation coefficient is known to be mathematically identical with coefficient η when one set of variables comprises of a number of binary indicator variables (i.e., the dummy variables also used for the calculation of η) and the other set includes only one continuous variable (Klecka 1980). However, the canonical correlation does not make the distinction between dependent and independent variable as it is the case for the η coefficient. The calculation of η is well aligned with the idea of mean comparisons of a continuous dependent variable (i.e., ability estimates) between groups that are defined by a categorical independent variable (i.e., distractor choices). However, in nested logit models, the relationship between ability and distractor choice is modeled vice versa: Distractor choice is modeled as a function of ability. Hence, we argue in favor of the canonical correlation because it does not suffer from this conceptual confusion, while simultaneously maintaining its potential for the detection of item-wise distractor discrimination.

In the context of this work, the canonical correlation is based on two sets of variables: (a) A matrix **X**_1_, including *k* binary indicator variables with an entry of 1 in the *k*th column and *v*th row when person *v* chose distractor *k* and zero otherwise, and (b) a vector **x**_2_ that includes the total scores for all participants who did not solve the item under consideration. Then, **r**_12_ is the column vector, including the correlations between each column from **X**_1_ and **x**_2_, and **H**_1_ is the Cholesky decomposition (Harville 2008) of the correlation matrix between all binary indicator variables in **X**_1_. With these terms in mind, the canonical correlation can be expressed as

(4) $$R_{CC}=d_{11},$$

with *d*_11_ is the only element from the **D** matrix resulting from a singular value composition (Harville 2008) of **W** = $r_{12}^{\prime }H_{1}^{-1}$. For the canonical correlation the same cut-offs for the detection of items with discriminatory distractors are suggested as it was the case above for Cohen’s ω*_G_* (small—0.10; medium—0.30; and large—0.50). Example-Item 1 had *R_CC_* = 0.01, whereas, Example-Item 2 had *R_CC_* = 0.34. This illustrates that the discriminatory power of distractors could also be detected by means of *R_CC_*.

### 1.3. Aim of the Current Study

In the current work, a thorough simulation study was undertaken to examine the potential of Cohen’s ω and *R_CC_* (as outlined above) to detect items for their potential to discriminate individuals with respect to their latent trait based on distractor choice behavior. To this aim, we first simulated conditions in which distractors did not possess discriminatory power with respect to the latent trait to assess the type-I-error of the used statistical indices (i.e., effect sizes passing the effect size threshold, when the population model did not include discriminatory distractors). Second, we simulated conditions based on a population model with discriminatory distractors to examine the power to detect items that are suitable for nested logit modeling. A final aim of this work is to illustrate the suggested item-analytical strategy by means of the data taken from Myszkowski and Storme (2018).

## 2. Simulation Study 

### 2.1. Method

#### 2.1.1. Data Generating Model

The data were simulated according to a 2PNL (Suh and Bolt 2010) in which the probability that person *j* solves item *i* is modeled by the following logistic model:

(5) $$P(x_{ij}=u|\theta_{j})=\frac{1}{1+e^{-\left(\beta_{i}+\alpha_{i}\theta_{j}\right)}}.$$

*u* is the correct option, *θ_j_* is the ability parameter, *β_i_* is the item difficulty parameter, and α*_i_* is the discrimination parameter. Then, in case that an item has not been solved the probability to choose distractor *v* among the set of the remaining *m_i_* distractors is modeled by the nominal response model with intercept parameters ζ*_iv_* and distractor discrimination parameters *λ_iv_*:

(6) $$P(x_{ij}=v|\theta_{j})=\left[1-P(x_{ij}=u|\theta_{j}\right)]\left[\frac{e^{\zeta_{iv}+\lambda_{iv}\theta_{j}}}{\sum_{k=1}^{m_{i}}e^{\zeta_{ik}+\lambda_{ik}\theta_{j}}}\right].$$

#### 2.1.2. Facets of the Simulation Design

Several factors were manipulated to allow a thorough investigation of the usefulness to detect items with discriminatory distractors for nested logit modeling:

1. Sample size (three levels): *N* = 100; *N* = 200; and *N* = 500.
2. Number of items (three levels): *I* = 10; *I* = 20; and *I* = 50.
3. Number of distractors (two levels): *D* = 3; and *D* = 7.
4. 2-PL difficulty (three levels): Moderate [*β_i_* ~ *U*(−0.15, 0.15)]; difficult [*β_i_* ~ *U*(−1.15, −0.85)]; and very difficult [*β_i_* ~ *U*(−2.25, −1.85)].
5. 2-PL discrimination (three levels): Low [*α_i_* ~ *U*(0.25, 0.55)]; moderate[*α_i_* ~ *U*(0.85, 1.15)]; and high [*α_i_* ~ *U*(1.60, 1.90)].
6. NRM discrimination parameters (four levels) are depicted in Table 2.

NRM intercepts ζ*_iv_* were further sampled for all design cells from a *U*(−1, 1) distribution. Further facets resulted from the used effect size threshold and the type of effect size (but these facets did not imply additionally generated datasets):

7. Effect size threshold (three levels): Small: Effect size > 0.10; moderate: Effect size > 0.30; and large: Effect size > 0.50.
8. Type of effect size (three levels): Cohen’s ω based on two ability groups; Cohen’s ω based on five ability groups; and the canonical correlation coefficient.

#### 2.1.3. Dependent Variables

The main dependent variable was: (1) The proportion of effect sizes that were larger than the effect size threshold. In addition, we examined the following dependent variables related to the empirical substance of the simulated datasets: (2) the proportion of distractors with relative choice frequencies smaller than 5%; (3) the proportion of missing effect sizes; (4) the proportion of missing effect sizes resulting from too many distractors with relative choice frequencies smaller than 5%; and (5) the proportion of ability groups occurring in the simulated data. For example, a value of 0.99 for Cohen’s ω based on five ability groups and in case of 10 simulated items implies that 0.99 × 5 × 10 = 4950 groups were simulated out of 5000 possible groups.

#### 2.1.4. Simulation Setup

All simulations and analysis were carried out by means of the statistical software R (R Core Team 2019). The simulation of the datasets was performed with the simdata() function included in the R package mirt (Chalmers 2012). The design for the dataset generation was based on crossing all facets of the simulation design (see 1. to 6. presented in Section 2.1.2). Hence, it was a sample-size × number-of-items × number-of-distractors × 2-PL-difficulty × 2-PL-discrimination × NRM-discrimination design with 3 × 3 × 2 × 3 × 3 × 4 = 648 cells. For each of these 648 cells, we generated 1000 datasets and aggregated the dependent variables across these datasets for each cell. All R code files and simulated data are available in the OSF repository for this work (https://osf.io/9tp8h/).

### 2.2. Simulation Results

#### 2.2.1. Type-I-Error Results

**Figure 2:**
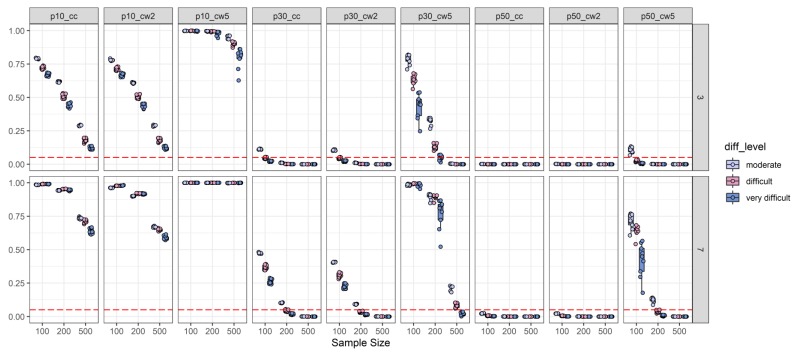
2PL-difficulty split of type-I-error analysis: Depiction of the type-I-error rate (y-axis) as a function of sample size (x-axis), number of distractors (three distractors = top-row; seven distractors = bottom-row), 2PL-difficulty (diff_level: Moderate vs. difficult vs. very difficult), and effect size measures combined with effect size thresholds (see explanation) (p10_cc = canonical correlation with a 0.10 threshold; p10_cw2 = Cohen’s ω based on two ability groups with a 0.10 threshold; p10_cw5 = Cohen’s ω based on five ability groups with a 0.10 threshold; p30_cc = canonical correlation with a 0.30 threshold; p30_cw2 = Cohen’s ω based on two ability groups with a 0.30 threshold; p30_cw5 = Cohen’s ω based on five ability groups with a 0.30 threshold; p50_cc = canonical correlation with a 0.50 threshold; p50_cw2 = Cohen’s ω based on two ability groups with a 0.50 threshold; p50_cw5 = Cohen’s ω based on five ability groups with a 0.50 threshold). The horizontal red dashed line represents the target type-I-error rate of 0.05.

Results with respect to type-I-error revealed a clear picture of findings. First, an effect size threshold of 0.10 appeared to be far too liberal regardless of any other design facet. In fact, for all cells in the design, the type-I-error rate for the 0.10 threshold was clearly above the conventional 0.05 level (see Figure 2). Second, the worst performance in terms of type-I-error rate was observed for Cohen’s ω based on five ability groups. This effect size measure reached only acceptable type-I-error rates for very specific conditions (see Figure 2). For example, with three distractors and a 0.30 threshold, ω_5_ was adequate only when the sample size was *N* = 500. Moreover, for seven distractors, a 0.30 threshold, and a sample size of *N* = 500 acceptable type-I-error rates were reached only for very difficult items. The best performance of Cohen’s ω based on five ability groups was found for the three-distractor condition and a threshold of 0.50 (i.e., only type-I-error for moderately difficult items was too large). However, both other effect size measures (Cohen’s ω based on two ability groups and the canonical correlation) yielded highly conservative type-I-error rates (i.e., type-I-error rates that are notably smaller than 0.05) when the effect size threshold was 0.50 regardless of any other design facet. Moreover, Cohen’s ω based on two ability groups and the canonical correlation coefficient yielded acceptable to conservative type-I-error rates with three distractors and a threshold of 0.30 when 2PL-difficulty was at least difficult. The same was observed for these two effect size measures for seven distractors, but only when the sample size was at least *N* = 200 (see Figure 2). Finally, we found that 2PL-difficulty was inversely related to type-I-error rates as in several simulated conditions moderate 2PL-difficulty resulted in the highest type-I-error rate (see Figure 2), whereas, the level of 2PL-discrimination and the number of items did not show any specific relationship with a type-I-error rate (see Appendix A). 

Based on these findings, we refrain from any power examinations for conditions with an effect size threshold of 0.10. It is further noteworthy that for all other effect size thresholds Cohen’s ω_2_ (85%) and *R_CC_* (84%) comparable numbers of cells with acceptable type-I-error rates resulted, whereas, Cohen’s ω_5_ displayed acceptable type-I-error rates only for 48% of the simulated cells. Thus, Cohen’s ω_5_ appeared to have only a very narrow range of scenarios in which this statistic is advisable for the detection of discriminatory distractors. Cohen’s ω_2_ and *R_CC_*, however, were found to function comparably well (see also Table 3). 

#### 2.2.2. Power Results

Prior to power analysis, all results for conditions that yielded unacceptable type-I-error rates were removed. Given that effect size measures are studied for their potential usefulness in the context of test-development, it is unlikely that other important item statistics, such as Goodman and Kruskal’s γ to test for rising selection ratios or *PB_DC_* would be ignored. Hence, we checked all conditions that had both acceptable type-I-error and sufficient power (i.e., power ≥ 0.80) for their power under additional boundary conditions. First, the power of effect size measures was reevaluated under the additional condition that the average γ is greater than 0.30. Second, another reevaluation of the power of effect size measures took *PB_DC_* as a boundary condition into account. Here we tested the additional condition that the average *PB_DC_* had to be smaller than −0.30. Table 3 displays the percentages of design cells with adequate empirical power with and without boundary conditions.

Across various conditions, the percentage of design cells with adequate power was highest for the canonical correlation (see Table 3). The only exception to this pattern was the moderate NRM discrimination condition paired with an effect size threshold of 0.50. However, in these conditions the best-performing statistic was ω_5_ and adequate power was only achieved for less than 10% of the design cells, which was still surpassed by *R_CC_* paired with a 0.30 threshold (see Table 3). Results indicated further, as expected, a positive relationship between NRM discrimination and empirical power. That is, the higher the NRM discrimination was in the data-generating model; the higher was the percentage of design cells with adequate power to detect discriminatory distractors (see Table 3). This pattern was rather robust across effect size thresholds and the used effect sizes. Restricting the findings to *R_CC_* as the overall best-performing statistic, however, revealed that power gains from *high* to *very high* NRM conditions were negligible (even non-existent when boundary conditions were taken into account) with a 0.30 threshold. Comparing further the *R_CC_* results between the 0.30 and the 0.50 threshold, independent of NRM discrimination, suggested that the power advantage of the lower 0.30 threshold as compared to the 0.50 threshold vanishes for *very high* NRM discrimination. The differences between power analyses without and with boundary conditions increased with the level of NRM discrimination. Generally, the overall impression of empirical power results was supported regardless of the presence of additional boundary conditions.

**Figure 3:**
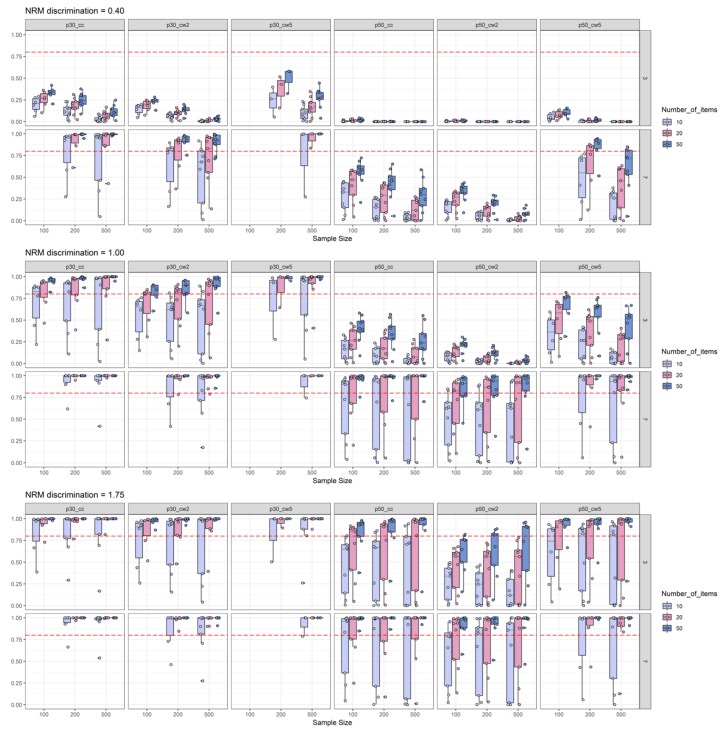
Number-of-items split of empirical power analysis: Depiction of the empirical power (y-axis) as a function of NRM discrimination (0.40 = top-row; 1.00 = middle-row; and 1.75 = bottom-row), sample size (x-axis), number of distractors (three distractors = top-row in each sub-plot; seven distractors = bottom-row in each sub-plot), number of items, and effect size measures combined with effect size thresholds. The horizontal red dashed line represents the target power level of 0.80. For more explanations, see Figure 2.

In Figure 3, the power simulation results are split according to NRM discrimination conditions between panels and according to the number of items. We found that empirical power was positively related to the number of items and the number of distractors, indicating that more items and more distractors increase empirical power to detect informative distractors. The simulation results might give further the impression that for most of the conditions, sample size was negatively related to empirical power. However, when comparing Figure 3 with Figure 5, it becomes clear that this impression occurs, due to the influence of the low 2PL discrimination conditions on sample-size-specific empirical power (i.e., there seems to be an interaction between sample size and 2PL discrimination level). Otherwise, some conditions revealed a positive relationship between empirical power and sample size. For example, for high NRM discrimination, three distractors, at least 20 items, and for a threshold of 0.30, empirical power was a positive function of sample size for both the canonical correlation coefficient and Cohen’s ω based on two ability groups (see Figure 3). For these conditions, empirical power also surpassed the 0.80 level for sufficient power. Moreover, detection of moderately discriminate distractors was possible by means of the canonical correlation, but only with seven distractors, at least 20 items and a threshold of 0.30. Under the same conditions, Cohen’s ω needed at least 50 items for sufficient power. Detection of discriminatory distractors with three distractor items required at least a high NRM discrimination with again the best findings for the canonical correlation that required at least 20 items for adequate power (as compared to at least 50 items for Cohen’s ω). 

**Figure 4:**
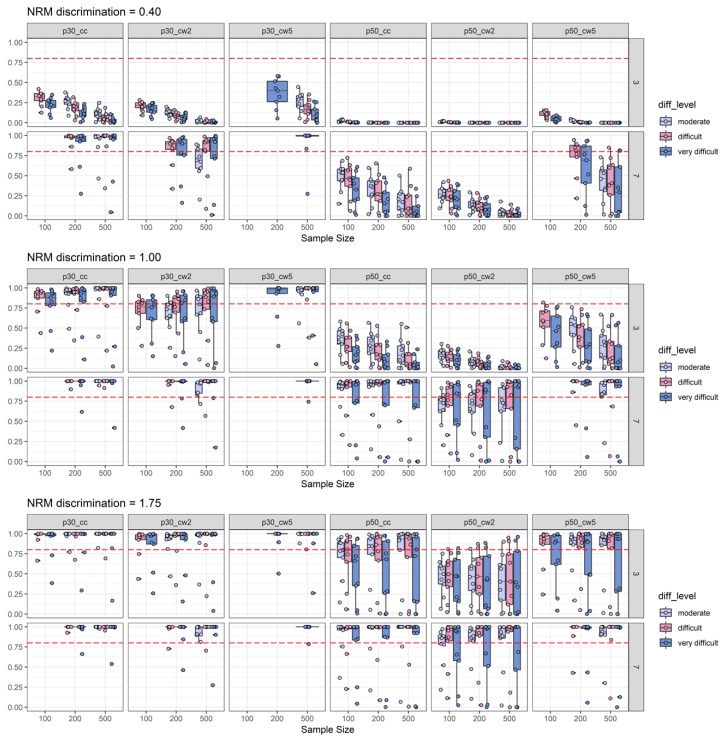
2PL-difficulty split of empirical power analysis: Depiction of the empirical power (y-axis) as a function of NRM discrimination, sample size (x-axis), number of distractors, 2PL-difficulty (diff_level: Moderate vs. difficult vs. very difficult), and effect size measures combined with effect size thresholds. The horizontal red dashed line represents the target power level of 0.80. For more explanations, see Figure 2 and Figure 3.

**Figure 5:**
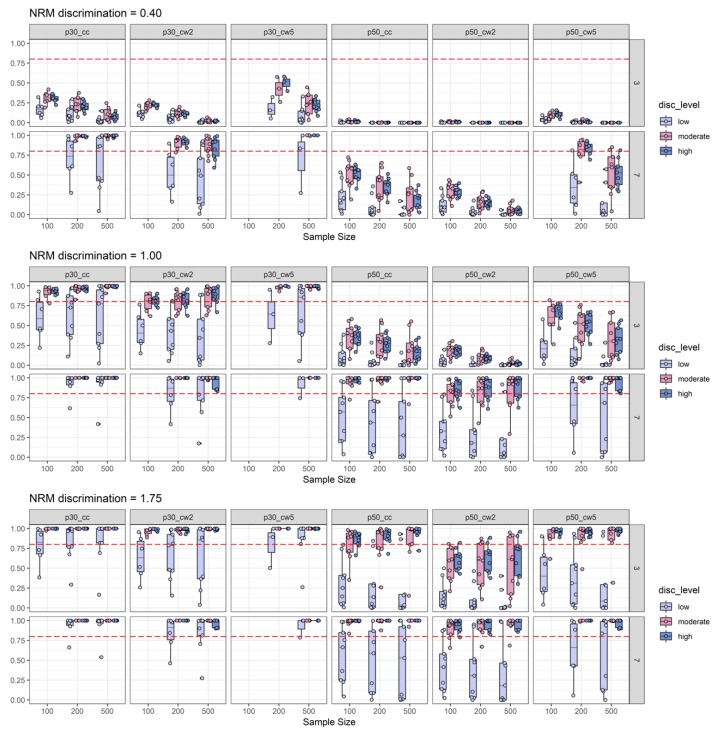
2PL-discrimination split of empirical power analysis: Depiction of the empirical power (y-axis) as a function of NRM discrimination, sample size (x-axis), number of distractors, 2PL-discrimination (disc_level: Low vs. moderate vs. high), and effect size measures combined with effect size thresholds. The horizontal red dashed line represents the target power level of 0.80. For more explanations, see Figure 2 and Figure 3.

In Figure 4, the boxes of the boxplots are depicted in different colors depending on 2PL difficulty. While recognizing that item easiness is negatively associated with the available data for participants choosing one of the distractors, this plot also suggests a picture in line with the idea that the effect sizes are subject to an upward bias. Here again, we suggest a cautious interpretation, because this impression was driven by low 2PL-discrimination conditions (see Figure 5) that were presented among the other findings for the varying difficulty conditions. Again, under these various 2PL difficulty conditions, the canonical correlation combined with a 0.30 threshold displayed the best findings with respect to empirical power across various conditions. The exceptions from this pattern can be inferred from Figure 5 to be caused by conditions in which 2PL discrimination was low (see the skyblue boxplots in the subplots for p30_cc). Hence, one might conclude that after a check of item-scale correlations, the canonical correlation combined with a 0.30 threshold seems to be the best choice for the task of pre-selecting items with discriminatory distractors from a pilot study (please note that this conclusion also takes type-I-error into account, because power was only examined for conditions with acceptable type-I-error rates). The detailed power findings presented in Figure 3, Figure 4 and Figure 5 replicated well under additional boundary conditions as it was the case for the results aggregated in Table 3. Appendix B provides detailed figures of power findings under boundary conditions. 

#### 2.2.3. Empirical Substance Examination

We further examined the empirical substance for each of the simulation conditions. First, the percentage of distractors with relative choice frequencies smaller than 5% increased with NRM discrimination (9.62%, 10.34%, 15.56%, and 21.33% for NRM discrimination levels of 0, 0.40, 1.00, and 1.75, respectively). The percentage of missing effect sizes, due to distractor choice frequencies < 5%, however, was rather small in conditions (the maximum was < 1% for zero and moderate NRM discrimination; and maximally 1.67% or 3.96% for NRM discrimination levels of 1.00 and 1.75, respectively). The overall percentage of missing effect sizes was then examined, and across all 648 cells of the simulation design, we found 17 cells with percentages of missing effect sizes larger than 1%. An amount of 1% of missing effect sizes implies, for example, that for 1000 replications and 10 items the number of missing effect sizes would be 100. The largest percentage of missing effect sizes was found to be 4% for the condition with *N* = 500, *I* = 10, *D* = 7, moderate 2PL-difficulty, high 2PL-discrimination, and the highest level of NRM discrimination. All 17 cells had in common that *D* = 7, 2PL-difficulty was moderate, 2PL-discrimination was high, and that NRM discrimination was at least high. For all other design cells, the percentage of missing effect sizes was below 1%, and for most of the cells, this percentage was zero or negligible. The minimal proportion of occurring group sizes in the data was 99.99% for ω_2_ in all conditions and 79.95% for ω_5_ in all conditions. Hence, ω_5_ was affected the most by empirical substance loss, which in turn might explain its inferior performance in the simulation study. 

#### 2.2.4. Discussion of Simulation Study Findings

In this simulation study, we thoroughly investigated the type-I-error rates and empirical power of *R_CC_*, ω_2_, and ω_5_ effect sizes to detect the discriminatory power of distractors. The power examination was also carried out under additional boundary conditions defined by effect sizes with a focus on solution behavior (i.e., γ and *PB_DC_*). The simulation was further flanked by an empirical substance investigation to reveal the amount of information loss when, for example, distractors are chosen by less than 5% of the participants or creation of ability groups did not result in the target number of groups. The aim of this simulation was twofold: (a) We wanted to identify the best-performing effect size for the detection of discriminatory distractors, and (b) we wanted to explore potential factors that influence type-I-error and empirical power.

Results suggested that *R_CC_* and ω_2_ yielded comparable performance with respect to type-I-error. *R_CC_* and ω_2_ displayed acceptable type-I-error for a far greater variety of simulated conditions as compared to ω_5_. Hence, ω_5_ was found to be clearly limited in its range of application. In terms of empirical power, however, it was found that *R_CC_* clearly outperformed ω_2_ in most of the simulated conditions with few design cells in which *R_CC_* and ω_2_ performed comparably well. In relation to this, it is further important that using *R_CC_* in combination with a 0.30 threshold yielded better empirical power findings in conditions with moderate or high NRM discrimination. For very high NRM discrimination conditions *R_CC_* combined with a 0.30 threshold and *R_CC_* combined with a 0.50 threshold were found to be comparable with respect to empirical power findings. Thus, for a wide range of simulated conditions in this study, *R_CC_* in combination with a 0.30 threshold would be the best choice.

A more fine-grained analysis of influencing factors on type-I-error and empirical power revealed that it is not generally recommended to use sample sizes of *N* = 100 for the detection of discriminatory distractors. Based on type-I-error findings, the sample size should be at least *N* = 200 when items include three distractors. When items include seven distractors, however, sample sizes below *N* = 500 cannot be recommended without further considerations, due to unacceptable type-I-error rates. In relation to this, it needs to be noted that type-I-error rates for a threshold of 0.50 were acceptable for all conditions when *R_CC_* as the generally best-performing effect size measure is used (i.e., ω_2_ also had acceptable type-I-error rates for all conditions with a 0.50 threshold, but did not perform on par with *R_CC_* with respect to power). However, in terms of empirical power, it is important to take further into account that 2PL-discrimination needs to be at least moderate and NRM discrimination had to be very high to yield largely acceptable detection power (with better findings for seven distractor items). When NRM discrimination is only high, detection of discriminatory distractors was only feasible for seven distractor items when at the same time 2PL-discrimination was at least moderate. Empirical power, with a 0.50 threshold to detect items with moderate NRM discrimination, was found to be unacceptable.

Importantly, the presented findings on the detection of ability-related discriminatory distractors suggest that there is no simple rule to increase the power analogous to experimental study planning (e.g., the more participants, the higher the power to detect a certain assumed mean difference between experimental groups). Simply raising sample size or the number of items (or even both) did not generally increase empirical power in the simulation. For example, with increasing sample sizes or increasing difficulty, it was found that variation in empirical power increased for low 2PL-discrimination conditions (see Figure 3, Figure 4 and Figure 5). Hence, 2PL-discrimination seems to be a precondition before power follows the commonly known “the-more-the-better” rule of thumb. In light of these specificities of the simulation findings, we, thus, recommend researchers to formulate their expectations (e.g., based on previous empirical studies) about a potential item pool with respect to important parameters that were found to be influential in this study, such as 2PL item difficulty (items should be difficult or very difficult) and 2PL item discrimination (items should have at least moderate 2PL discrimination) and run their own customized simulation to guide scale development. For example, scale development must be efficient sometimes, and it could be the case that only *N* = 150 participants are available for a first examination of the ability-related discriminatory power of distractors. Then, with the simulation code of this study as a starting point (the code is openly available at https://osf.io/9tp8h/), it is possible to explore different thresholds (i.e., also thresholds between 0.30 and 0.50) with respect to type-I-error and empirical power in combination with other characteristics (e.g., number of items or number of distractors) to choose the best design for scale development. Most likely, a design with *N* < 100 will not be applicable which prevents usage of the suggested approach at very early stages of scale development in which items are tested for clear instructions or the wordings of item content (Johanson and Brooks 2010).

## 3. Empirical Illustration

### 3.1. Method

#### 3.1.1. Dataset

The studied dataset was taken from Myszkowski and Storme (2018). This dataset includes *N* = 499 participants from a French business school (undergraduates; 285 females and 214 males; age: *M* = 20.70, *SD* = 0.93). All participants worked on the last series of Raven’s Standard Progressive Matrices (SPM-LS) without any imposed time limit. The instructions further encouraged the participants to provide a response even when they were unsure about the correct solution (Myszkowski and Storme 2018). Thus, no missing data are present in this dataset. The SPM-LS consists of twelve items with seven distractors each. Hence, this dataset closely mimics the design in the simulation study above with *N* = 500, *I* = 10, and *D* = 7. 

#### 3.1.2. Analytical Strategy

This empirical illustration will apply the effect size measures introduced in this work. Based on the findings of the simulation study, we calculated *R_CC_* as effect size with a threshold of 0.30 because it displayed acceptable type-I-error rates and reasonable empirical power under comparable conditions as given for the given dataset in the simulation study above. In addition, the number of distractors with relative choice frequencies < 0.05, *PB_DC_* and γ were calculated. Finally, we re-estimated the 2PL-parameters to facilitate interpretation of the findings in connection with the simulation study presented above.

### 3.2. Results and Discussion

The findings presented in Table 4 revealed that for items 1 to 5 distractor choice frequencies were too sparse to use the *R_CC_*. As expected, this sparsity of distractor frequencies was associated with 2PL-difficulty estimates. These five items were indeed among the easiest items according to the estimates in Table 4. Moreover, the estimates, in particular those for items 1 to 5, were much higher as compared to the difficulties simulated above. In fact, only items 10 to 12 were found to be in the range of simulated 2PL-difficulty values used above. The values for items 8 and 9 were closer to the moderate difficulty level used in the simulation, whereas, the estimates for items 6 and 7 were clearly easier. The 2PL-discrimination estimates, however, were inside the range of the simulation study and were even higher for several items. The latter observation is particularly important, because even for the detection of moderate NRM discrimination it was found that *R_CC_* had adequate power levels with seven distractors and a sample size of *N* = 500 (which are conditions resembling the Myszkowski-Storme dataset). Given that 2PL-discrimination was identified in the simulation as an important influencing factor on the detection power, one could reasonably assume that higher 2PL-discrimination can compensate for lower 2PL-difficulty as compared to the used simulation setup. This reasoning applies particularly to item 6, which was found to have a much larger 2PL-discrimination parameter estimate as compared to the values used in the simulation (and a much lower difficulty estimate). Nonetheless, caution is needed when interpreting these findings with parameter estimates outside the simulated values.

Analysis of distractor effect size measures revealed five items (6, 8, 9, 10, and 12) with canonical correlation coefficients > 0.30. Hence, we would suggest that the items flagged for distractors with discriminatory power by means of the canonical correlation are most likely the ones driving the reliability gain at the low-ability range, as reported by Myszkowski and Storme (2018). Moreover, these items are expected to fit a nested logit modeling approach well in a larger sample.

To secure these observations, we further calculated the *PB_DC_* for items 6 to 12 to examine if the correct solution was associated with higher ability levels as compared to choosing one of the distractors. In addition, γ was used to check the rising selection ratio property. Table 4 displays the average *PB_DC_* across all distractors for each of the items. These values ranged from moderate to large effect sizes implying rather well-functioning distractors in this regard. Importantly, some items displayed comparable *R_CC_* values (items 9 and 10), but clearly varying *PB_DC_* values. This highlights the importance to study ability-related discrimination of distractors and discrimination with respect to solution behavior at the same time. *PB_DC_* and also γ focus on solution behavior, but *R_CC_* focus on distractors that are more often chosen by participants with higher ability levels as compared to other distractors (i.e., not in comparison to the correct solution). These aspects of discrimination are not necessarily expected to covary. This is further illustrated by the *R_CC_* and *PB_DC_* findings for item 11, which had the lowest *R_CC_* value, but the second strongest *PB_DC_* (see Table 4).

The average γ findings were in the range from small to large, with an average γ smaller than 0.30 for items 11 and 12. This highlights that for an item not all boundary conditions might be fulfilled (in these cases average *PB_DC_* was below −0.30, but γ was not larger than 0.30). To decide if this pattern is problematic for the detection of ability-related discriminatory distractors, new simulations to understand the interplay of *PB_DC_* and γ as boundary conditions are clearly needed. Hence, results for items 11 and 12 should also be treated with caution. These findings largely support the feasibility of nested logit modeling with its primary focus on solution behavior and distractor choices as additional information used for ability estimation.

## 4. Overall Discussion

In our study, we suggest an item-analysis procedure to detect items with potentially discriminating distractors that can be used for trait estimation in models, such as nested logit models which take both accuracy and distractor choices into account. We thoroughly examined the usefulness of different effect sizes for distractor discrimination by a simulation study, and illustrated our findings by an application to an empirical dataset with participants who worked on the short form of Raven’s Progressive matrices test. As such, our analysis had a different focus as compared to traditional distractor analyses which are usually concerned with distractor choice frequency and variants of biserial correlations to evaluate distractor quality (Gierl et al. 2017; Haladyna and Downing 1993; Attali and Fraenkel 2000; Haladyna 2004). Instead, Cohen’s ω was examined as an effect size that can potentially reveal interactions between ability groups and distractor choice frequencies, which are indicative of the discriminatory power of distractors for the trait under consideration. As a second effect size, the canonical correlation was studied as a measure for the detection of the discriminatory power of item distractors in terms of the latent trait variable. The simulation revealed that in contrast to Cohen’s ω, the canonical correlation coefficient seems to be most promising for the task of detecting items with discriminatory distractors.

### Limitations

This work is limited to the simulation conditions chosen. For example, Myszkowski and Storme (2018) highlight the importance of taking item guessing into account. That is, they suggest relying on the 3PNL instead of relying on the 2PNL as we used in the simulation. Likewise, the 4PNL was not studied here to reduce the complexity of the simulation design. We argue that for a starting point to understand mechanisms behind item selection based on distractor effect size measures, the design was already rather complex. Future studies are clearly required in this regard.

In the empirical illustration, the suggested effect sizes for distractor discrimination were flanked by the average *PB_DC_*, and the average γ and this approach seems to be most promising. In particular, the *PB_DC_* can further reveal if choosing the correct solution is more strongly related to the ability as compared to any other distractor. This is crucial for a model that puts solution behavior in the first place. Moreover, average γ ensures that the assumption of rising selection ratios holds for a set of candidate items which is further useful to guide item pre-selection. In our simulation, we found that using these two statistics as boundary conditions is useful, but for simplicity, these two item statistics were studied in isolation. Obviously, other more complex item-analysis strategies could be used in which also a combined cut-off for both statistics is used. It is further possible to consider scenarios in which item-selection is carried out in multiple consecutive steps, and the current work does not shed much light into the question which statistic should be consulted first (e.g., testing discrimination in the sense of item-scale correlations first, testing for rising selection ratios second, and screening for ability-related discriminatory power of distractors as a final step). 

## 5. Conclusions

Overall, the potential of nested logit models to construct measures with higher measurement precision at lower ability levels by means of exactly the same items is highly attractive not only for cognitive ability constructs as intelligence, but especially for measures that need to be very short and efficient. However, the long tradition of, for instance, figural matrix items and available theories with respect to solution behavior, item-, and distractor generation principles seem to be a key requisite for the construction of such a measure. The canonical correlation as an effect size for distractor discrimination seems to be a promising statistical tool for pre-selecting useful items for scale construction in this regard. In fact, the canonical correlation can be interpreted analogously to classical item-scale correlations (i.e., test developers can rely on a familiar 0.30 cut-off). Moreover, it fits the idea of item response models in which observable behavior (i.e., distractor choice) is modeled as a function of ability.

## Figures and Tables

**Table 1:** Distractor specific statistics for Example-item 1 and Example-item 2.

Distractor	Relative Choice Frequency < 0.05	*PB_D_*	*PB_DC_*	ω*_D_*	γ
	Item 1/Item 2	Item 1/Item 2	Item 1/Item 2	Item 1/Item 2	Item 1/Item 2
Distractor 1	0/0	−0.18/−0.21	−0.54/−0.58	0.57/0.68	1/1
Distractor 2	0/0	−0.29/−0.08	−0.56/−0.47	0.55/0.39	1/1
Distractor 3	0/0	−0.13/−0.36	−0.50/−0.68	0.58/0.97	1/1

Relative choice frequency < 0.05 = Number of distractors with a relative choice frequency below 0.05; *PB_D_* = point-biserial correlation for the contrast between participants who chose *D* vs. participants who chose any other option (including the correct option) with respect to test performance; *PB_DC_* = point-biserial correlation for the contrast between participants who chose *D* vs. participants who chose the correct option with respect to test performance; ω*_D_*—Haladyna-Downing approach (Haladyna and Downing 1993) = Cohen’s ω based on choice frequencies restricted to *D* as a function of 5 ability groups based on equi-distant quantiles; γ = Goodman-Kruskal γ for the relationship between test performance based on all other items and the probability to choose the correct response as estimated based on a 2 × *J* contingency table (with *J* is the number of possible performance scores) which has been suggested by (Love 1997) as an index for the evaluation of rising selection ratios.

**Table 2:** NRM discrimination parameters used in the simulation study.

Distractor	Level 1—Zero	Level 2—Moderate	Level 3—High	Level 4—Very High
	3 distractors/7 distractors	3 distractors/7 distractors	3 distractors/7 distractors	3 distractors/7 distractors
λ*_i_*_1_	0.00/0.00	−0.40/−1.20	−1.00/−3.00	−1.75/−5.25
λ*_i_*_2_	0.00/0.00	0.00/−0.80	0.00/−2.00	0.00/−3.50
λ*_i_*_3_	0.00/0.00	0.40/−0.40	1.00/−1.00	1.75/−1.75
λ*_i_*_4_	-/0.00	-/0.00	-/0.00	-/0.00
λ*_i_*_5_	-/0.00	-/0.40	-/1.00	-/1.75
λ*_i_*_6_	-/0.00	-/0.80	-/2.00	-/3.50
λ*_i_*_7_	-/0.00	-/1.20	-/3.00	-/5.25
NRM discrimination (step size)	0.00	0.40	1.00	1.75

See DeMars for a comparable approach to simulate items with discriminating distractors according to the NRM (DeMars 2003). NRM discrimination (step size): This is the step size between the consecutive λ*_i_* parameters that can be used as a general indicator of NRM discrimination.

**Table 3:** Percentages of cells in the simulation design with adequate type-I-error rate and empirical power.

	Threshold = 0.30			Threshold = 0.50		
	*R_CC_*	ω_2_	ω_5_	*R_CC_*	ω_2_	ω_5_
**Adequate type-I-error rate**						
All	69	**70**	25	**100**	**100**	72
**Adequate power**						
**NRM Discrimination = 0.40**						
All	**23**	17	5	0	0	**7**
*M*(γ) > 0.30	**17**	3	4	-	-	**6**
*M*(*PB_DC_*) < −0.30	**16**	3	4	-	-	**3**
**NRM Discrimination = 1.00**						
All	**61**	45	23	**35**	24	23
*M*(γ) > 0.30	**39**	31	16	**25**	17	14
*M*(*PB_DC_*) < −0.30	**41**	30	16	**24**	15	15
**NRM Discrimination = 1.75**						
All	**65**	62	26	**65**	41	54
*M*(γ) > 0.30	**38**	**38**	14	**41**	25	36
*M*(*PB_DC_*) < −0.30	40	**41**	15	**44**	24	40

Percentages are rounded to integers. The threshold-specific best-performing statistic is highlighted in bold. When two effect sizes performed equally well, both were highlighted. The total number of cells for each of the respective levels of NRM discrimination was 162 (please note that the cells for checking type-I-error rates had zero NRM discrimination). *M*(γ) > 0.30: In addition to adequate empirical power (≥0.80) the boundary condition that the average of Goodman-Kruskal’s γ to check rising selection ratios had to be greater than 0.30. *M*(*MP_DC_*) < −0.30: In addition to adequate empirical power the boundary condition that *PB_DC_* to check discrimination between item solvers and participants who chose a certain distractor had to be smaller than −0.30. Frequencies in bold font refer to the best performing effect sizes under the respective threshold conditions.

**Table 4:** Distractor choice frequency, 2PL-parameter estimates, and distractor effect size measure findings on the Myszkowski-Storme dataset.

Item	Number of Distractors with Relative Choice Frequency < 0.05	2PL-Difficulty	2PL-Discrimination	*R_CC_*	*M*(*PB_DC_*) ^2^	*M*(γ) ^3^
Item 1	6	1.32	0.85	NA ^1^	NA ^1^	NA ^1^
Item 2	7	3.56	2.01	NA ^1^	NA ^1^	NA ^1^
Item 3	6	2.07	1.69	NA ^1^	NA ^1^	NA ^1^
Item 4	6	4.11	4.10	NA ^1^	NA ^1^	NA ^1^
Item 5	7	5.51	4.97	NA ^1^	NA ^1^	NA ^1^
Item 6	5	2.13	2.38	0.46	−0.38	0.48
Item 7	4	1.23	1.55	0.28	−0.36	0.40
Item 8	1	0.50	1.61	0.34	−0.47	0.65
Item 9	3	0.40	1.27	0.34	−0.39	0.37
Item 10	1	−0.70	2.20	0.36	−0.61	0.77
Item 11	1	−0.82	1.51	0.21	−0.48	0.21
Item 12	1	−0.91	1.14	0.31	−0.43	0.23

^1^ Distractor effect size measures were not calculated when the number of distractors with relative choice frequency < 0.05 exceeded a value of five (i.e., when only one distractor remained for analysis). ^2^ The average of all *PB_DC_* values for all available distractors of an item is reported—the lower the average *PB_DC_*, the better. ^3^ The average of all γ values for all available distractors of an item is reported—the higher the average γ, the better. The R code to reproduce the findings in this table can be found in Appendix C.

**Figure A1:**
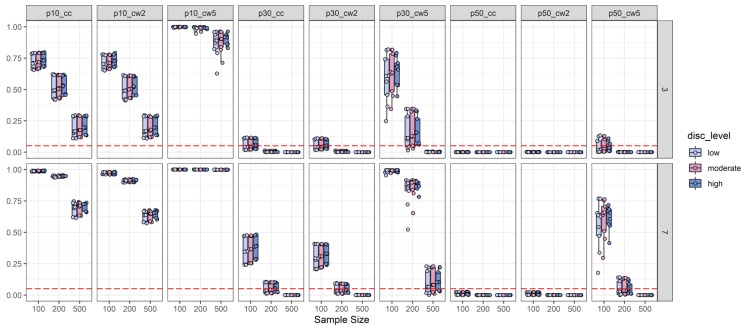
2PL-discrimination split of type-I-error analysis: Depiction of the type-I-error rate (y-axis) as a function of sample size (x-axis), number of distractors (three distractors = top-row; seven distractors = bottom-row), 2PL-discrimination (disc_level: Low vs. moderate vs. high), and effect size measures combined with effect size thresholds. The horizontal red dashed line represents the target type-I-error rate of 0.05. For more explanations, see Figure 2.

**Figure A2:**
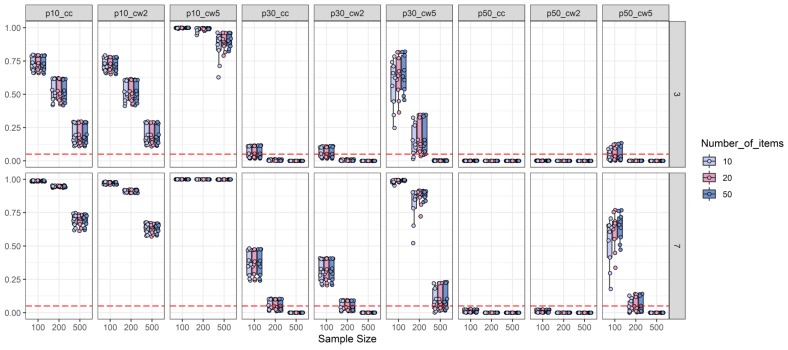
Number-of-items split of type-I-error analysis: Depiction of the type-I-error rate (y-axis) as a function of sample size (x-axis), number of distractors (three distractors = top-row; seven distractors = bottom-row), number of items and effect size measures combined with effect size thresholds. The horizontal red dashed line represents the target type-I-error rate of 0.05. For more explanations, see Figure 2.

**Figure A3:**
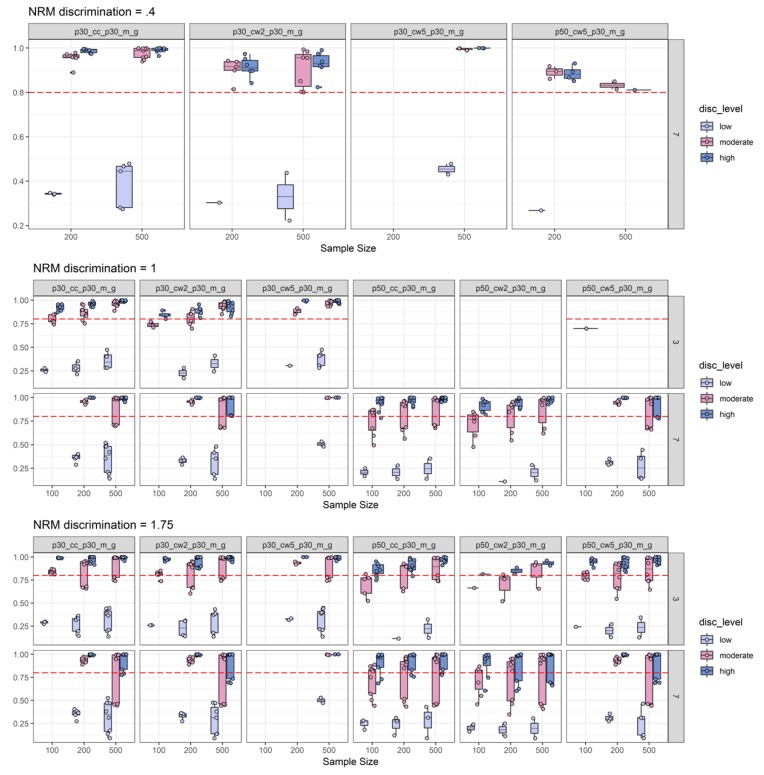
2PL-discrimination split of empirical power analysis: Depiction of the empirical power (y-axis) under the boundary condition that the average γ is greater than 0.30. Power is depicted as a function of NRM discrimination, sample size (x-axis), number of distractors, 2PL-discrimination, and discrimination effect sizes combined with effect size thresholds (p30_cc_p30_m_g = canonical correlation with a 0.30 threshold; p30_cw2_p30_m_g = Cohen’s ω based on two ability groups with a 0.30 threshold; p30_cw5_p30_m_g = Cohen’s ω based on five ability groups with a 0.30 threshold; p50_cc_p30_m_g = canonical correlation with a 0.50 threshold; p50_cw2_p30_m_g = Cohen’s ω based on two ability groups with a 0.50 threshold; p50_cw5_p30_m_g = Cohen’s ω based on five ability groups with a 0.50 threshold). The horizontal red dashed line represents the target power level of 0.80. For more explanations, see Figure 2 and Figure 3.

**Figure A4:**
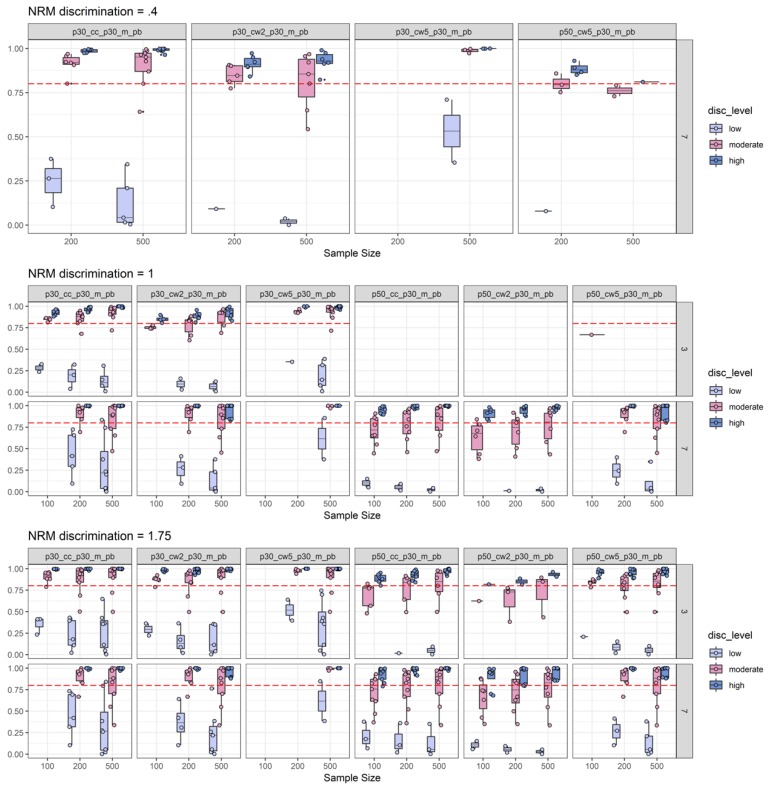
2PL-discrimination split of empirical power analysis: Depiction of the empirical power (y-axis) under the boundary condition that the average *PB_DC_* is smaller than −0.30. Power is depicted as a function of NRM discrimination, sample size (x-axis), number of distractors, 2PL-discrimination, and discrimination effect sizes combined with effect size thresholds (p30_cc_p30_m_pb = canonical correlation with a 0.30 threshold; p30_cw2_ p30_m_pb = Cohen’s ω based on two ability groups with a 0.30 threshold; p30_cw5_ p30_m_pb = Cohen’s ω based on five ability groups with a 0.30 threshold; p50_cc_ p30_m_pb = canonical correlation with a 0.50 threshold; p50_cw2_ p30_m_pb = Cohen’s ω based on two ability groups with a 0.50 threshold; p50_cw5_ p30_m_pb = Cohen’s ω based on five ability groups with a 0.50 threshold). The horizontal red dashed line represents the target power level of 0.80. For more explanations, see Figure 2 and Figure 3.

**Figure A5:**
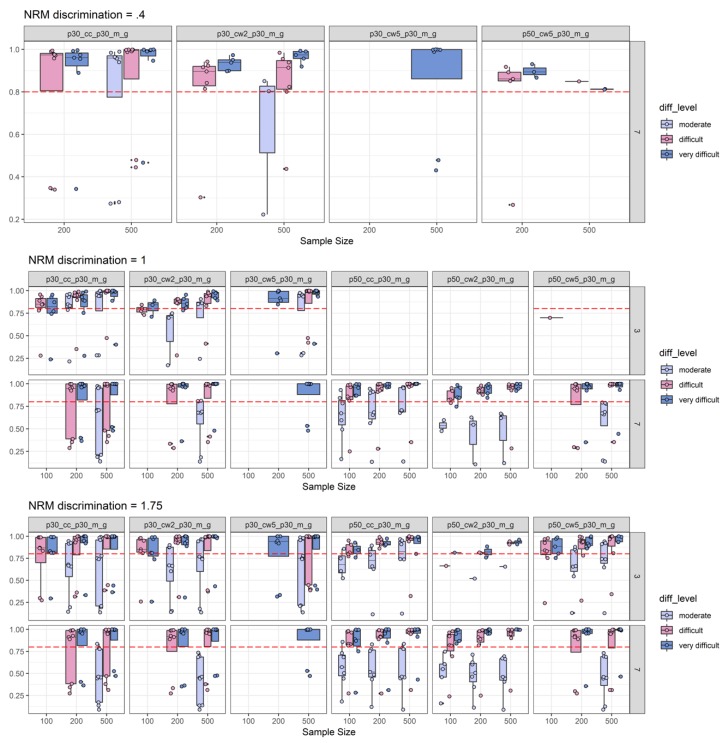
2PL-difficulty split of empirical power analysis: Depiction of the empirical power (y-axis) under the boundary condition that the average γ is greater than 0.30. Power is depicted as a function of NRM discrimination, sample size (x-axis), number of distractors, 2PL-difficulty, and discrimination effect sizes combined with effect size thresholds. The horizontal red dashed line represents the target power level of 0.80. For more explanations, see Figure 2 and Figure 3.

**Figure A6:**
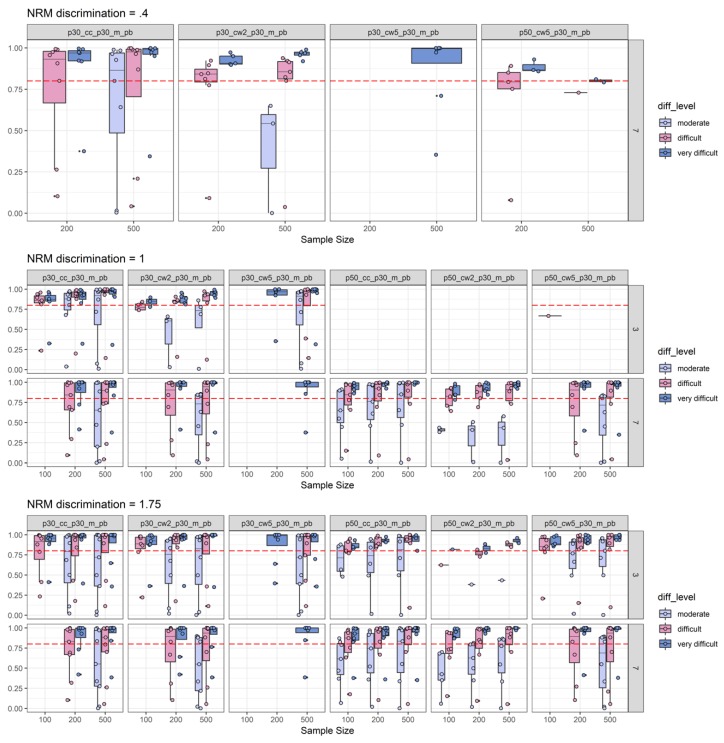
2PL-difficulty split of empirical power analysis: Depiction of the empirical power (y-axis) under the boundary condition that the average *PB_DC_* is smaller than −0.30. Power is depicted as a function of NRM discrimination, sample size (x-axis), number of distractors, 2PL-difficulty, and discrimination effect sizes combined with effect size thresholds. The horizontal red dashed line represents the target power level of 0.80. For more explanations, see Figure 2 and Figure 3.

**Figure A7:**
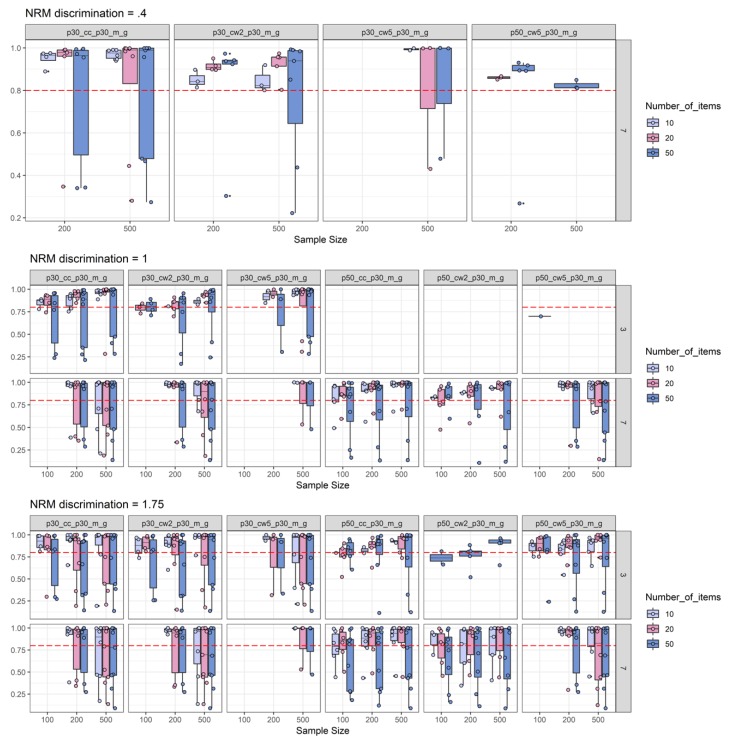
Number-of-items split of empirical power analysis: Depiction of the empirical power (y-axis) under the boundary condition that the average γ is greater than 0.30. Power is depicted as a function of NRM discrimination, sample size (x-axis), number of distractors, number of items, and discrimination effect sizes combined with effect size thresholds. The horizontal red dashed line represents the target power level of 0.80. For more explanations, see Figure 2 and Figure 3.

**Figure A8:**
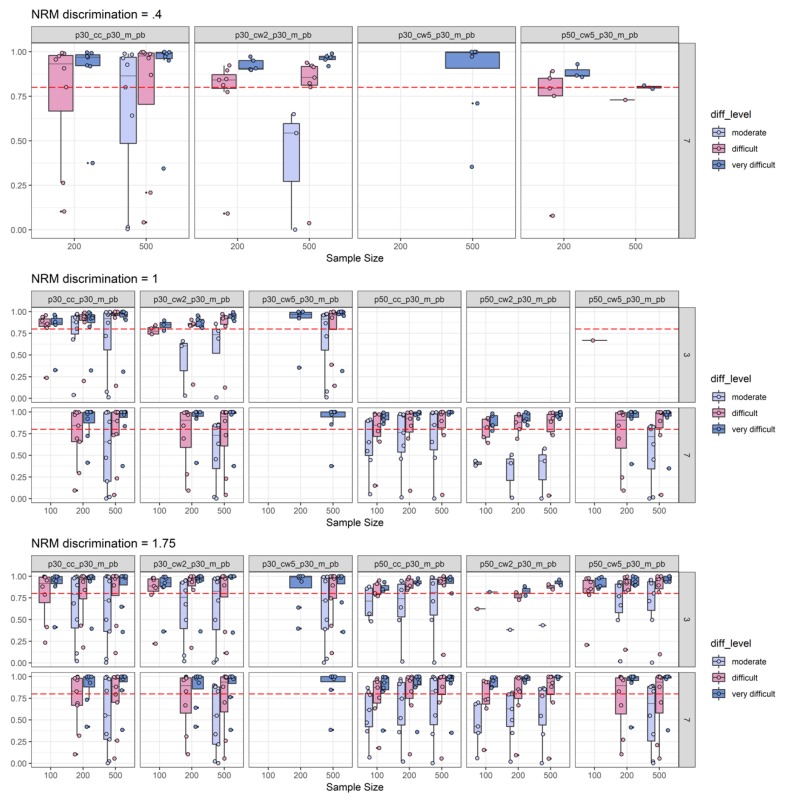
Number-of-items split of empirical power analysis: Depiction of the empirical power (y-axis) under the boundary condition that the average *PB_DC_* is smaller than −0.30. Power is depicted as a function of NRM discrimination, sample size (x-axis), number of distractors, and discrimination effect sizes combined with effect size thresholds. The horizontal red dashed line represents the target power level of 0.80. For more explanations, see Figure 2 and Figure 3.

## Appendix A

In this appendix, further simulation results on the type-I-error rate are displayed in Figure A1 and Figure A2.

## Appendix B

In this appendix, further simulation results on the empirical power under boundary conditions are displayed. Figure A3 shows the findings for average γ as a boundary condition when the boxes in the boxplots are grouped by the levels of simulated 2PL discrimination. The power findings replicated well even with this boundary condition. Only low 2PL discrimination conditions were associated with clearly decreasing levels of power far below the target level of 0.80. The same observation was made with average *PB_DC_* as a boundary condition (see Figure A4). 2PL discrimination was found to be the strongest influencing factor on these examinations of boundary conditions. For 2PL difficulty, a similar pattern was revealed with the lowest power for moderately difficult items (in some cases even dropping below the 0.80 target level) and the highest power for very difficult items (see Figure A5 and Figure A6). When structuring the boxes in the boxplots according to the number of items, it was further revealed that the number of items was inversely related to the dispersion of power results (see Figure A7 and Figure A8). With ten items power findings were pretty homogenous for all studied effect size measures, but for 50 items, the power results were strongly scattered.

## Appendix C

In this appendix, the R code to reproduce the analysis for the Myszkowski-Storme dataset is presented. Two additional packages were used: Psych (Revelle 2018) for the scoring of the multiple-choice items, and mirt (Chalmers 2012) for estimation of the 2PL parameters. The complete R code, including also the simulation study is available in the OSF repository: https://osf.io/9tp8h/.

### load data
#
### downloaded from
# https://data.mendeley.com/datasets/h3yhs5gy3w/1
dataset <- read.csv(“dataset.csv”, stringsAsFactors=FALSE)

### install required packages (if needed)
### remove # to make this code run
#install.packages(c(“psych”,”mirt”))

### get results function
### includes also the effect size measures that were
### studied in the simulation and also more useful
### descriptive statistics
get_results <- function(data,keys=“sim”){
  ### keys for scoring
  if(length(keys)==1){keys <- rep(0,ncol(data))}else{
    keys <- keys
  }
  ### quantiles for two ability groups
  p2 <- .5
  ### quantiles for five ability groups
  p5 <- c(.2,.4,.6,.8)
  ### load psych library
  require(psych)
  ### score all items
  scored <- score.multiple.choice(key=keys,data=data,score=F)
  ### ability groups
  abil2.c <- rep(0,nrow(scored))
  for(i in 1:length(p2)){
    if(i < length(p2)){  
      abil2.c[rowSums(scored)>quantile(rowSums(scored),p=p2[i])
              & rowSums(scored)<=quantile(rowSums(scored),p=p2[i+1])] <- i
    }else{abil2.c[rowSums(scored)>quantile(rowSums(scored),p=p2[i])] <- i
    }    
  }
  ### ability groups
  abil5.c <- rep(0,nrow(scored))
  for(i in 1:length(p5)){
    if(i < length(p5)){  
      abil5.c[rowSums(scored)>quantile(rowSums(scored),p=p5[i])
              & rowSums(scored)<=quantile(rowSums(scored),p=p5[i+1])] <- i
    }else{abil5.c[rowSums(scored)>quantile(rowSums(scored),p=p5[i])] <- i
    }    
  }
  ### list distractors with relative frequency < .05
  rf05 <- list()
  for(j in 1:ncol(data)){
    rf05[[j]] <- table(data[,j][data[,j]!=keys[j]])/length(data[,j])<.05

  }
  ### general Cohen’s w, 2 ability groups
  chi_g2 <- list()
  cw_g2 <- list()
  tab_c2_l <- list()
  zero_columns2 <- list()
  for(k in 1:ncol(data)){
    tab_c2 <- matrix(table(data[,k][data[,k]!=keys[k]],abil2.c[data[,k]!=keys[k]])[!rf05[[k]],],ncol=length(unique(abil2.c[data[,k]!=keys[k]])))
    zero_columns2[[k]] <- colSums(tab_c2)==0
    tab_c2 <- tab_c2[,colSums(tab_c2)>0]
    tab_c2_l[[k]]<-tab_c2
    if(sum(!rf05[[k]])>=2){chi_g2[[k]] <- chisq.test(tab_c2)}else{
      chi_g2[[k]] <- NA
    }
    ### Cohen’s w - general
    if(sum(!rf05[[k]])>=2){cw_g2[[k]] <- sqrt(sum(((chi_g2[[k]]$observed/sum(tab_c2)-chi_g2[[k]]$expected/sum(tab_c2))^2)/(chi_g2[[k]]$expected/sum(tab_c2))))}else{
      cw_g2[[k]] <- NA
    }
  }
  ### general Cohen’s w, 5 ability groups
  chi_g5 <- list()
  cw_g5 <- list()
  tab_c5_l <- list()
  zero_columns5 <- list()
  for(k in 1:ncol(data)){
    tab_c5 <- matrix(table(data[,k][data[,k]!=keys[k]],abil5.c[data[,k]!=keys[k]])[!rf05[[k]],],ncol=length(unique(abil5.c[data[,k]!=keys[k]])))
    zero_columns5[[k]] <- colSums(tab_c5)==0
    tab_c5 <- tab_c5[,colSums(tab_c5)>0]
    tab_c5_l[[k]]<-tab_c5
    if(sum(!rf05[[k]])>=2){chi_g5[[k]] <- chisq.test(tab_c5)}else{
      chi_g5[[k]] <- NA
    }
    ### Cohen’s w - general
    if(sum(!rf05[[k]])>=2){cw_g5[[k]] <- sqrt(sum(((chi_g5[[k]]$observed/sum(tab_c5)-chi_g5[[k]]$expected/sum(tab_c5))^2)/(chi_g5[[k]]$expected/sum(tab_c5))))}else{
      cw_g5[[k]] <- NA
    }
  }
  ### canonical correlation
  can_cor <- list()
  ncol_mmat <- list()
  for(k in 1:ncol(data)){
    ncol_mmat[[k]] <- if(sum(!rf05[[k]])>=2){ncol(model.matrix(rowSums(scored[scored[,k]==0,-1])~-1+factor(data[,k][scored[,k]==0]))[,!rf05[[k]]])}else{
      NA
    }
    can_cor[[k]] <- if(sum(!rf05[[k]])>=2){cancor(rowSums(scored[scored[,k]==0,-k]),model.matrix(rowSums(scored[scored[,k]==0,-1])~-1+factor(data[,k][scored[,k]==0]))[,!rf05[[k]]])$cor}else{
      NA
    }
  }
  ### point-biserial coefficient PB_DC
  pb_dc <- list()
  ### Goodman-Kruskal gamma
  gkg <- list()
  gkg_tab <- list()
  ### start loop
  for(v in 1:ncol(data)){
    pb_dc_d <- list()
    gkg_d <- list()
    gkg_tab_d <- list()
    ### function to calculate
    ### Goodman-Kruskal gamma
    ### taken from here:
    ### https://stat.ethz.ch/pipermail/r-help/2003-March/030835.html
    goodman <- function(x,y){
      Rx <- outer(x,x,function(u,v) sign(u-v))
      Ry <- outer(y,y,function(u,v) sign(u-v))
      S1 <- Rx*Ry
      return(sum(S1)/sum(abs(S1)))}
    ### start loop
    non_key <- unique(data[,v])[!unique(data[,v])%in%keys[v]]
    for(w in non_key){
      MDC <- mean(rowSums(scored)[data[,v]%in%c(keys[v],w)])
      SDC <- sd(rowSums(scored)[data[,v]%in%c(keys[v],w)])
      MD <- mean(rowSums(scored)[data[,v]%in%w])
      PD <- mean(data[,v]%in%w)
      PC <- mean(data[,v]%in%keys[v])
      ### r-PB_D
      ### r-PB_DC
      pb_dc_d[[w]] <- (MD-MDC)/SDC*sqrt(PD/PC)
      ### Goodman-Kruskal gamma
      score_other_items <- factor(rowSums(scored[,-v]))
      tab_gkg_d <- table(data[data[,v]%in%c(keys[v],w),v],score_other_items[data[,v]%in%c(keys[v],w)])
      ### exclude ability levels with zero frequency
      tab_gkg_d <- tab_gkg_d[,colSums(tab_gkg_d)>0]
      gkg_d[[w]] <- goodman(as.numeric(colnames(tab_gkg_d)),
                            tab_gkg_d[as.numeric(rownames(tab_gkg_d))%in%keys[v],]/colSums(tab_gkg_d))
      gkg_tab_d[[w]] <- tab_gkg_d
    }
    pb_dc[[v]] <- pb_dc_d
    gkg[[v]] <- gkg_d
    gkg_tab[[v]] <- gkg_tab_d
  }

  ### return results
  res <- list(rf05 = rf05,
              tab_c2_l = tab_c2_l, zero_columns2 = zero_columns2,
              tab_c5_l = tab_c5_l, zero_columns5 = zero_columns5,
              cw_g2 = cw_g2, cw_g5 = cw_g5,
              can_cor = can_cor,
              pb_dc = pb_dc,
              gkg = gkg,
              gkg_tab = gkg_tab,
              ncol_mmat = ncol_mmat)
  return(res)
}

### frequencies of distractor usage
### including correct response
apply(dataset,2,table)

### load psych library
library(psych)

### score all items
scored <- score.multiple.choice(key=c(7,6,8,2,1,5,1,6,3,2,4,5),data=dataset,score=F)

### does choosing a certain other distractor
### imply better overall scores?
#
### run suggested distractor analysis
ms_res<-get_results(dataset,keys = c(7,6,8,2,1,5,1,6,3,2,4,5))

### show Results for Table 4
#
### show for which items the distractor choice frequencies were 
### below 5%:
ms_res$rf05
### Items 1 to 5 have too many distractors with response frequencies
### below 5%.
#
### get 2PL parameters from mirt
library(mirt)
est_test2pl <- mirt(scored, 1, itemtype=“2PL”)
### show results
coef(est_test2pl)
# a1 = 2PL-discrimination
# d  = 2PL-difficulty
#
### canonical correlation findings
ms_res$can_cor[6:12]
### check boundary conditions
#
### average pb_dc
lapply(ms_res$pb_dc,function(x)mean(unlist(x),na.rm=T))[6:12]
### average gamma
lapply(ms_res$gkg,function(x)mean(unlist(x),na.rm=T))[6:12]
		
